# Proteomic profiling of lymphocytes in autoimmunity, inflammation and cancer

**DOI:** 10.1186/1479-5876-12-6

**Published:** 2014-01-07

**Authors:** Jiebai Zhou, Zhitu Zhu, Chunxue Bai, Hongzhi Sun, Xiangdong Wang

**Affiliations:** 1 Department of Pulmonary Medicine, Zhongshan Hospital, Fudan University, Shanghai Medical College, Shanghai, China; 2 Shanghai Respiratory Research Institute, Shanghai, China; 3 Center for Cancer Molecular and Cellular Therapies, The First Affiliated Hospital of Liaoning Medical University, Liaoning, China

**Keywords:** Proteomics, Lymphocyte, Autoimmune, Allergic inflammation, Cancer

## Abstract

Lymphocytes play important roles in the balance between body defense and noxious agents involved in a number of diseases, e.g. autoimmune diseases, allergic inflammation and cancer. The proteomic analyses have been applied to identify and validate disease-associated and disease-specific biomarkers for therapeutic strategies of diseases. The proteomic profiles of lymphocytes may provide more information to understand their functions and roles in the development of diseases, although proteomic approaches in lymphocytes are still limited. The present review overviewed the proteomics-based studies on lymphocytes to headlight the proteomic profiles of lymphocytes in diseases, such as autoimmune diseases, allergic inflammation and cancer, with a special focus on lung diseases. We will explore the potential significance of diagnostic biomarkers and therapeutic targets from the current status in proteomic studies of lymphocytes and discuss the value of the currently available proteomic methodologies in the lymphocytes research.

## Introduction

Lymphocytes play central and pivotal roles in immune defense mechanisms against pathogens and controls of the immune responses, including protective responses against pulmonary pathogens and lung diseases, e.g. asthma, sarcoidosis, idiopathic pulmonary fibrosis, or alveolitis [1-4]. The emergence of genomics, proteomics, and bioinformatics allows defining gene expression and/or protein profile in lymphocytes to furthermore understand interactions between the components within targeted signaling network, even though a number of challenges still exist. The protein expression and post-translational modifications profiled by proteomics can explore functional interactions between proteins and develop disease-specific biomarkers by integrating with clinical informatics [5-7]. Proteomic tools and strategies are used to elucidate mechanisms and function of lymphocyte signaling and biology, including glycoprotein, phosphoprotein, membrane protein, single protein, quantitative protein and organelle [8]. Localizations, post-translational modifications, interactions, or turnovers of proteins produced by lymphocytes are summarized in Table 1. The present review overviewed the proteomics-based studies on lymphocytes to headlight the proteomic profiles of lymphocytes in diseases, such as autoimmune diseases, allergic inflammation and cancer, with a special focus on lung diseases. We will explore the potential significance of diagnostic biomarkers and therapeutic targets from the current status in proteomic studies of lymphocytes and discuss the value of the currently available proteomic methodologies in the lymphocytes research.

**Table 1:** Categories of study design and techniques in lymphocyte proteomics

**Ref.**	**Disease/Activity**	**Total identified proteins**	**Samples**	**Country**	**Proteomic methods**
10	IL-12 regulated CD4+ T cells differentiation	42	Blood	Finland	2-DE
					MS
13	T cell proliferation	17	Blood	Hong Kong, China	2-DE
					MALDI-TOF MS
23	Mucosal tolerance	11	Lymph node	Netherlands	2D-PAGE
					MALDI-TOF MS
27	T-cell vaccination (TCV)	11	Activated mouse ovalbumin-specific T cells	P. R. China	2-DE
					Q-TOF MS
28	Vogt-Koyanagi-Harada (VKH) syndrome	30	Blood	United States of America	LC-MS/MS
29	Rheumatic heart disease (RHD)	3	Blood	Brazil	2-DE
			Cardiac tissue		MALDI-TOF MS
32	Tyrosine phosphorylation	3	Primary human lymphocyte	United States of America	2D-PAGE
37	Immunosuppression	10	E6-1 cell line	Spain	2D-PAGE
38	Colitis	26	Lymph node	P. R. China	2D-PAGE
					MS
45	Pseudomonas aeruginosa sepsis	11	Blood	P. R. China	2-DE
					MALDI-TOF MS
46	DNA repair capacity (cell-cycle checkpoint-related protein)	8	Blood	United States of America	Reverse-phase protein lysate microarray assay
49	Asthma	25	Blood	South Korea	2D-PAGE
					MALDI-TOF MS
50	Asthma	13	Blood	Taiwan, China	2D-PAGE
					LC/MS

### Autoimmune diseases

The production of auto-antibodies from T- and B- lymphocytes were associated with lymphocyte-mediated immune responses [9]. Forty-two proteins were differentially expressed in human CD4^+^ T cells during the differentiation of naïve CD4^+^ T cells into Th1 cells induced by interleukin (IL)-12, of which 22 and 20 were up- and down- regulated respectively [10], as shown in Table 2. In this particular study, macrophage migration inhibitory factor and programmed cell death 4 involved in cell rescue, defense, death and ageing were up-regulated. Macrophage migration inhibitory factor contributes to pro-inflammatory, anti-inflammatory and oxidoreductase activities, while its polymorphisms were associated with T helper cell-related disorders [11,12]. Down-regulation of p21-activated kinase 2, GTPase Cdc42, and a heat shock family member mortalin 2 may promote lymphocyte survival and protection from programmed cell death.

**Table 2:** Dominant proteins associated with T cell activities

**Ref.**	**Activity**	**Protein**	**Expression**	**Functions**
10	T cell differentiation	Macrophage migration inhibitory factor (MIF)	Up-regulated	Cell resque, defense, death, ageing
		Programmed cell death 4 (pdcd4)	Up-regulated	Cell resque, defense, death, ageing
		p21-activated kinase 2 (Pak2)	Down-regulated	Signal transduction
		Cdc42	Down-regulated	Signal transduction
		Mortalin 2 (Mot-2)	Down-regulated	Protein destination
13	T cell proliferation	Peroxiredoxin 1	Up-regulated	A scavenger of reactive organic hydroperoxides
		Proteasomes	Up-regulated	A large multisubunit proteolytic complex that involved in an ATP/ubiquitin-dependent non-lysosomal proteolytic pathway
		Triosephosphate isomerase (TIM)	Up-regulted	Enzyme of metabolic pathways like converting glyceraldehyde-3-phosphate to dehydroxyacetone phosphate in the glycolytic pathway
		Galectin-1, Chain A	Down-regulated	An anti-inflammatory agent which triggers homeostatic signals to shut off T cell effector functions such as cell proliferation, cell death and cytokines synthesis, cell adhesion and inflammation
23	T cell supression	Haptoglobin	Up-regulated	Anti-inflammatory activities
		Nonintegrin 67 kDa laminin receptor (LR)	Down-regulated	Lymphocyte migration
		MRP8	Up-regulated	Marker proteins for activated or recruited phagocytes
27	T cell activation	Calreticulin (CRT)	Identified	Damage-associated molecular pattern molecules, inducing immunogenic apoptosis
		Endoplasmic reticulum protein (ERp57)		
		Vimentin		

Proteins involved in the T cell proliferation were defined during the suppression of PHA-treated T cells isolated from healthy donors, of which 13 proteins, e.g. peroxiredoxin-1, proteasomes and triosephospohate isomerase increased, while 4 like galectin-1 and Chain A reduced [13], as listed in Table 2. The identified differentially expressed proteins in lymphocytes were mainly involved in energy metabolism [14], anti-inflammatory pathways [15], oxidative stress [16] and protein breakdown [17], while a few were related to immune cell migration [18] and molecular chaperone [19]. T cell activation was inhibited by special inhibitors, e.g. cyclosporine A, polysaccharopeptide, or medical fungal products, through the alteration of proteins associated with the cell proliferation, including proteasomes, triosephospohate isomerase, galectin-1, Chain A, or perxiredoxin-1, respectively. Most of the altered proteins have functional significance in protein degradation [20], antioxidant pathway, energy metabolism [21], and immune cell regulation [22].

The intranasal delivery of auto-antigen could effectively induce mucosal tolerance and suppression of autoimmune diseases. Proteomic profiles of cervical lymph nodes were investigated to identify markers of the tolerant state 24, 48 and 72 h after nasal administration of antigen [23]. Of them, haptoglobin, nonintegrin 67 kDa laminin receptor (also known as P40-8), or Calgranulin A (MRP8) were related to the tolerance of immune through altering migration/mobility of antigen presenting cells and/or T cells [24,25] and anti-inflammatory activities [26]. The application of irradiated T cells as T-cell vaccination showed preventive effects and experimental treatment in autoimmune diseases, through induction and production of anti-lymphocytic antibodies and inhibition of T-cell proliferation.

Proteomics is used not only for the identification and validation of biomarkers, but also for the characterization of the antigen specificity. Eleven antigens were identified in mouse T cells activated by ovalbumin and recognized by anti-T cell antibodies after the animals were immunized with ovalbumin-specific T cells, of which damage-associated molecular pattern molecules were found to be dominant [27]. γ-radiation was found to induce the immunogenic apoptosis of activated T cells. Exposed/secreted damage-associated molecular pattern molecules, e.g. calreticulin, endoplasmic reticulum protein 57, and vementin, played an important role in T-cell vaccination therapy (Table 2).

T cells are considered as a major player in Vogt-Koyanagi-Harada (VKH) syndrome, an autoimmune disorder, which mainly affects systemic melanocytes in eyes, menings, ears, skin, or hairs. Membrane proteins in CD4^+^ T cells from patients with active VKH syndrome were analyzed with a label-free quantitative proteomic strategy to identify disease-associated proteins as compared with healthy individuals [28]. 102 significantly altered peptides were identified and corresponded to 64 proteins, of which 30 had 1.5-fold alterations, as summarized in Table 3. The expression of CD18 and AT-hook transcription factor decreased and then further validated in an additional group of patients with VKH syndrome, major players in the pathogenesis of the syndrome. Molecular mimicry between group A streptococcus antigens and host proteins was investigated in B and T cells from patients with rheumatic heart disease with the major manifestation of rheumatic fever due to streptococcus pharyngitis. Three proteins in heart infiltrating and peripheral T cells isolated from the valvular tissue from patients with chronic rheumatic heart disease were identified as T cell-targeted proteins, e.g. disulfide isomerase ER-60 precursor, 78 kD glucose-regulated protein precursor, and vimentin, with coverage of 45, 43, and 34%, respectively [29]. They were believed similar to antigens involved in T cell-mediated autoimmune responses in rheumatic fever/rheumatic heart diseases.

**Table 3:** **The differentially expressed proteins identified in CD4**^+^**T cells between active VKH patients and normal individuals**

**Identified proteins**	**Expression**	**Number of peptides**	**Function**
Integrin β	Up-regulated	Singe	Protein binding
Coronin-2A	Up-regulated	Singe	Protein binding
C19orf2 protein	Up-regulated	Singe	Protein binding
Isoform 4 of Dystrophin	Up-regulated	Singe	Structural constitute of cytoskeleton, muscle; protein, calcium ion, actin, zinc ion binding
Synaptonemal complex protein 2	Up-regulated	Singe	DNA binding
Hypothetical protein LOC345651	Up-regulated	Singe	Protein binding
Isoform 1 of Solute carrier family 22 member 11	Up-regulated	Singe	Sodium-independent organic anion transporter activity
UBC; UBB ubiquitin and ribosomal protein S27a precursor	Up-regulated	Singe	Structural constituent of ribosome
PRA1 family protein 3	Up-regulated	Singe	Protein binding
Actin, cytoplasmic 1	Up-regulated	Singe	Structural constituent of cytoskeleton; protein, ATP, nucleotide binding
Probable G-protein coupled receptor 179 precursor	Up-regulated	Singe	Metabotropic glutamate, GABA-B-like receptor activity
Heat shock 70 kDa protein 9, mitochondrial precursor	Up-regulated	Singe	Unfolded protein binding; ATP, nucleotide binding
ELMO2 protein	Up-regulated	Singe	Binding
Isoform 1 of Peripherin	Down-regulated	Singe	Structural molecule activity
ATP synthase subunit α, mitochondrial precursor	Down-regulated	Singe	Protein, ATP, nucleotide, metal ion binding; hydrogen-transporting ATPase/ATP synthase activity; hydrolase, transporter activity
Calnexin precursor	Down-regulated	Singe	Calcium ion, sugar binding; unfolded protein binding
Integrin β-2 precursor	Down-regulated	Singe	Protein, protein kinase binding; receptor activity
Vimentin	Up-regulated	Two or more	Structural constituent of cytoskeleton; protein binding; structural molecule activity; oxygen transporter activity
Keratin, type I cytoskeletal 10	Up-regulated	Two or more	Structural constituent of epidermis; structural molecule activity
ATP synthase subunit β, mitochondrial precursor	Up-regulated	Two or more	Protein, ATP, nucleotide, metal ion binding; hydrogen-transporting ATPase/ATP synthase activity; hydrolase, transporter activity; hydrogen-exporting ATPase activity; nucleotide-triphosphatase activity
Actin, aortic smooth muscle	Up-regulated	Two or more	Structural constituent of cytoskeleton; protein, ATP, nucleotide binding
Keratin, type II cytoskeletal 1	Up-regulated	Two or more	Structural constituent of cytoskeleton; protein, sugar binding; receptor activity
Uncharacterized protein ALB	Up-regulated	Two or more	Protein, water, toxin, pyridoxal phosphate, drug, fatty acid, oxygen, copper ion, DNA, metal ion binding; carrier, antioxidant activity
Isoform ASF-1 of Splicing factor, arginine/serine-rich 1	Down-regulated	Two or more	Protein, nucleotide, RNA binding

### Allergic inflammation

Lymphocytes and their products, together with other leukocytes, contribute to the initiation and perpetuation of the inflammatory response, through the alterations of protein profiles [30]. Protein tyrosine phosphorylation was suggested to play a critical role in regulation of gene transcription, cell proliferation, differentiation, cytoskeletal organization, and survival in lymphocytes during the development of tissue remodeling in chronic inflammation induced by allergen [31]. Protein profiles associated with tyrosine phosphorylation were investigated in Daudi, Burkitt’s lymphoma-derived B cell lines, and BJAB, B cell lymphoma-derived B cell lines, using a battery of Src homology 2 domain probes, and similar patterns of tyrosine phosphorylation for the Fyn and phosphatidylinositol-3-kinase Src homology 2 were found but not in Crk Src homology 2 [32]. The fingerprinting of proteins associated with signal transduction pathways in response to IL-2 or interferon (IFN)-γ in human lymphocytes demonstrated that IFN-γ could induce STAT2 phosphorylation, IL-2-activated phosphatidylinositol-3-kinase phosphoprotein, or both, involved in phosphorylation of transcription factor Jak-1 by anti-phosphotyrosine immunoprecipitations and proteomic analysis [33]. Chromatin-associated regulatory factors play central roles in the regulation of cell proliferation, differentiation, senescence, and death in lymphocyte. Proteomic analysis of chromatin-associated proteins demonstrated that transcription and replication factors varied in human B cells (P493-6 cells) expressing c-Myc in a tetracycline-repressible manner [34].

Proteomic profiles in lymphocytes were analyzed under challenges with concanavalin A-stimulated mouse spleen cells cultured with or without cyclosporine A and re-stimulated in the presence or absence of concanavalin A, to monitor the response of protein profiles to immunosuppressions and activations [35]. Cyclosporine A was found to activate and program the transcription and translation of a large number of genes in T cells without further reactivation, once the immunosupressor and the activator were removed. Cyclosporine A could induce down-regulation of immune responses through the calcineurin-dependent dephosphorylation of nuclear factors in activated T cells, which was interfered by new and specific synthesized polypeptides without affecting the expression of CD44 and CD69 and early tyrosine phosphorylation-associated protein profiles [36]. Of 111 protein spots altered in human T cells cultured with the potent immunosuppressant rapamycin, 70 increased and 41 decreased, of which annexin V and Ro/SSA antigen increased and α-enolase and laminin receptor 1 decreased [37].

**Figure 1:**
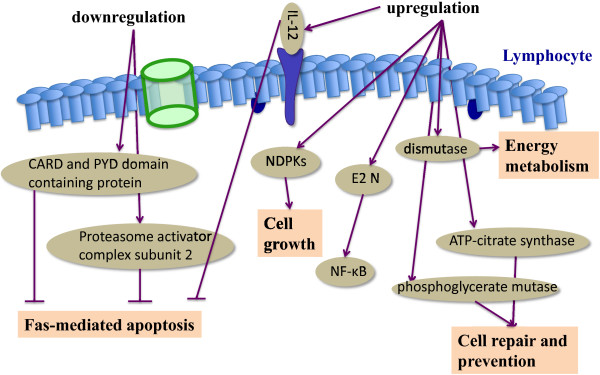
**Altered protein profile of lymphocytes from rats with colitis.** The altered proteins of lymphocytes in colitis fell into the following groups: apoptosis-related proteins, proteins associated with cell growth, differentiation and signal transduction, inflammation factors, proteins associated with metabolism and oxidative stress response. IL-12, interleukin-12; NDPKs, nucleoside diphosphate kinases; E2 N, ubiquitin-conjugating enzyme; NF-κB, nuclear factor κB.

There is increasing evidence to show that altered proteomic profiles of lymphocytes could dominate the development of allergic inflammation. For example, altered protein profiles of lymphocytes were investigated in an antigen-specific model of colitis induced by colonic administration of trinitrobenzene sulfonic acid/ethanol [38]. Of 1,100 protein spots, 26 proteins had more than at least two-fold differences between colitis and control, among which 17 up-regulated and 9 down-regulated, including CARD and PYD domain containing protein, proteasome activator complex subunit 2, IL-12 p40 precursor, nucleoside diphosphate kinase, ubiquitin-conjugating enzyme E2N, myeloid-related protein-14, ATP-citrate synthase, phosphoglycerate mutase and dismutase. The altered proteins served as regulators of the cell cycle and cell proliferation [39], signal transduction factors [40,41], inflammatory factors [42], apoptosis-related proteins [43,44] and metabolic enzymes, as illustrated in Figure 1. Alterations of protein profiles in lymphocytes in allergic diseases differed from those in serious diseases and bacterial infection [45]. Altered protein profiles in severe burn and Pseudomonas aeruginosa sepsis were found to be associated with the folding, assembling, transportation and degradation of proteins, signal transmission, inflammation, immunization, energy metabolism, the proliferation, differentiation and apoptosis of cells, including Cofilin, peptidyl-prolyl cis-trans isomerase cyclophilin A, ubiquitin, nucleoside diphosphate kinase, glutamate dehydrogenase, selenium binding protein I, beta-actin, peroxiredoxin-6, annexin I, actin-3 and cellular retinoic-acid binding protein.

### Cancer

The innate DNA repair capacity (DRC) is the central in controlling multistage carcinogenesis, and suboptimal DRC in peripheral lymphocytes was suggested as a cancer susceptibility marker and a functional link between DNA damage sensing, cell-cycle checkpoint, and DNA repair. Levels of *in vitro* cell-cycle checkpoint-related protein expressions in stimulated lymphocytes could predict DRC levels. The host-cell reactivation assay and the reverse-phase protein lysate microarray assay demonstrated that the DRC was significantly correlated with expression levels of cell-cycle checkpoint-related proteins, e.g. cyclin dependent kinase inhibitor (p27), cell cycle regulator (cyclin D1), DNA damage sensor (ataxia-telangiectasia mutated), and p53-negative regulator (mouse double minute 2 homolog), induced by benzo[a]pyrene diol epoxide-adducts [46]. Ataxia-telangiectasia mutated was identified as the most important predictor of DRC, followed by mouse double minute 2 homolog and p27, a population-based *in vitro* evidence that cell-cycle checkpoint-related proteins may play essential roles in the regulation of DNA repair in unaffected human peripheral blood lymphocytes. CD8^+^ T cells are considered as the major players in immune responses against cancer, with the cytotoxic activity and capability to release various mediators. Studies on network and functional characterization of proteins specific to resting or activated human CD8^+^ T cells provide new insight into the signaling pathway of CD8^+^ T cell activation with anti-CD3 and anti-CD28 mAb in the presence of IL-2 [47]. The altered expression of 35 proteins were mainly related with cell proliferation, metabolic pathways, antigen presentation, intracellular signal transduction pathways, as well as identification of six unknown proteins and predicted from genomic DNA sequences specific to resting or activated CD8^+^ T cells.

### Lung diseases

#### Asthma

**Figure 2:**
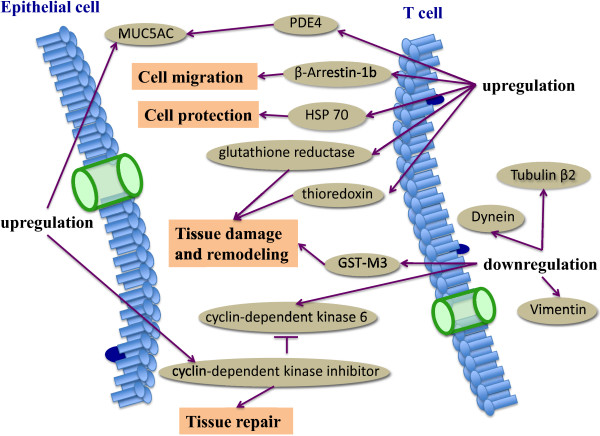
**Altered protein profiles of T cells from asthma patients.** PDE4, hydrolyzing cyclic adenosine monophosphate and/or cyclic guanosine monophosphate, was associated with the MUC5AC expression in human airway epithelial cells. β-Arrestin was suggested to play a role in T cell migration. Glutathione reductase, GST-M3 and thioredoxin were correlated with the antioxidant capacity. HSP-70 protects the cells and tissues from the deleterious effects of numerous mediators, reactive oxygen species, or tumor necrosis factor-α. Cytoskeletal proteins, including Dynein, Vimentin, Tubulin β2, might illustrate the functional changes in the T cells. Cyclin-dependent kinase was related to an abnormal repair process that might contribute to airway inflammation and remodeling. PDE, phosphodiesterase; HSP, heat shock protein; GST, glutathione S transferase.

Asthma is a complex disorder characterized by airway inflammation, goblet cell hyperplasia with mucus hypersecretion, and hypersponsiveness to various nonspecific stimuli. CD4^+^ T lymphocytes, especially Th2 lymphocytes, play an important role in the initiation, progression and persistence of asthma [48]. The expression of 13 proteins increased and 12 decreased in T lymphocytes from asthmatic patients, as compared to the healthy [49]. Among them, phosphodiesterase 4C, heat shock protein (HSP)-70, protein tyrosine phosphatase, β-arrestin-1b, thioredoxin-like 2, LLR protein, glutathione reductase, major histocompatibility complex class I antigen, epidermal growth factor receptor, pyrroline 5-carboxylate reductase isoform, and FLJ25770 protein increased, while dynein, cytoplasmic light intermediate polypeptide 2, vimentin, zinc finger protein 76, tubulin β_2_, T-cell receptor β-chain, cyclin-dependent kinase 6, pyridoxal kinase, phenylalanine hydroxylase, glutathione S-transferase-M3, and tetratricopeptide repeat-containing protein decreased. Of identified proteins, increased mRNA expression of phosphodiesterase 4C and thioredoxin-2 and decreased mRNA of glutathione S-transferase-M3 were further confirmed by RT-PCR in the large population of asthmatic patients. Figure 2 demonstrates some of the identified proteins and their role in asthma. To investigate the changes of proteins in T lymphocytes of asthmatic patients after no typical therapy (uncontrolled) to typical therapy (controlled) over 3 months, 7 proteins increased and 6 decreased in CD4^+^ T lymphocytes from uncontrolled asthmatic patients, including HSP-70, HSP-90, fibrinogen β chain, tropomyosin 3, ATP-dependent DNA helicase II, β-actin, vimentin, Rho GDP dissociation inhibitor beta, enolase 1, calreticulin precursor, tyrosine 3-monooxygenase/tryptophan 5-monooxygenase activation protein (YWHA), or peroxiredoxin 2, as compared to those from controls [50]. Two studies showed that HSP-70 increased and vimentin decreased in asthmatics and uncontrolled asthmatics. HSP is a ubiquitous, abundant, and conserved protein and synthetic rate of HSP increased in response to cellular stress, to protect the cells and tissues from the deleterious effects of numerous mediators, reactive oxygen species, or tumor necrosis factor-α. Other studies suggested that HSP is correlated with the severity of asthma exacerbation [51,52]. Vimentin, as a kind of cytoskeletal proteins and a type III intermediate filament protein normally expressed in cells of mesenchymal origin, attaches to the nucleus, endoplasmic reticulum, and mitochondria to control the shape, motility, and migration of mesenchymal cells [53]. The cytoskeletal changes in T lymphocytes of asthma patient may contribute to enhanced activity of proinflammatory and immune cells, reduced antioxidant defenses, functional changes in the T lymphocyte of asthma patient, or abnormal repair process responsible for airway inflammation and remodeling during damage to the epithelium. Such panel of altered proteins associated with the pathogenesis of asthma could be potential biomarkers and therapeutic targets in asthma.

#### Idiopathic Pulmonary Fibrosis (IPF)

IPF is a devastating disease characterized by a progressive distortion of the alveolar architecture and replacement by fibrotic tissue and extracellular matrix deposition. There are still no effective therapies for IPF which results in chronic respiratory failure and death associated with autoimmunity, though mechanism of autoimmunity involvement in the pathogenesis of IPF remains unclear. The expression of 51 proteins increased and 38 decreased in lungs of patients with sporadic IPF, as compared with healthy controls [54]. Up-regulated expression of markers for the unfolded protein response, HSP, and DNA damage stress markers indicated a chronic cell stress-response in IPF lungs. Down-regulated proteins in IPF included antioxidants, annexin family, and structural epithelial proteins, related with redox imbalance, epithelial cell injury, extensive fibrotic reaction, and increased susceptibility of IPF patients to infection. Proteomic analysis also identified for the first time the presence of anti-periplakin (PPL) antibodies in serum and brochoalveolar lavage fluid in patients with IPF [55]. PPL is a small protein from the plakin family, closely related to envoplakin and localized to demosomes and intermediate filaments [56]. PPL is constitutively expressed in both bronchial and alveolar epithelia and the intracellular distribution of PPL is modified in the hyperplastic alveolar epithelium of fibrotic lungs. PPL interacts with intermediate filaments in the cell and is required for the reorganization of keratin intermediate filaments network at the wound edge of simple epithelia cell monolayers and for the correct closure of an experimental wound [57]. The C-terminal domain of PPL can be linked to several intracellular proteins, such as protein kinase B ATK1/PKB [58] or FcγRI [59], leading to changes in cellular functions. Anti-PPL antibodies have the potential to interfere with alveolar repair and are associated with the severity of the disease, indicating that the abnormal responses of autoimmune contribute directly to the pathogenesis of IPF.

#### Lung cancer

Lung cancer is the most common cause of cancer-related mortality in the world, resulting in an overall 5-year survival rate of approximately 15% [60]. Proteome components related to therapeutic responses and targeted proteins in lung cancer cells and tissues were identified to define potential biomarkers with diagnostic, prognostic, and predictive values or drug-targeted candidates. To achieve a difference in the management of lung cancer, short of preventing its development, it is important to diagnose it early while it is measurable yet presymptomatic. The development of specific and sensitive diagnostic biomarkers from biological fluids, such as sputum, blood, or exhaled breath, should improve early detection strategies, monitoring of disease progression, treatment response, and surveillance for recurrence.

### Proteomic methodologies

Proteins are separated from cells and then subjected to complexity reduction, either via chromatographic or electrophoretic fractionation, or via enrichment of desired components, like membrane proteins, a particular sub-cellular component, or specific protein complex separation via affinity purification. Proteins can be identified via proteolysis and analysis of resultant peptides in mass spectrometer (MS), followed by database-searching. MS is an indispensable core of proteomic technologies and allows highly sensitive and high-throughput identification of proteins, post-translational modifications, or protein interaction [61,62]. Two-dimensional gel electrophoresis for separation of complex protein samples from blood, lymph nodes or cell lines, coupled with MS for protein identification, can analyze protein expression patterns for profiling lymphocyte proteomics, as shown in Table 1. A large variation of MS technologies is currently available, evolved from electrospray ionization and matrix-assisted laser desorption/ionization (MALDI) to a new generation of mass analyzers and complex multistage instruments [such as hybrid quadrupole time-of-flight (Q-TOF)] [61,63]. The MS technologies in lymphocyte proteomics include MALDI-TOF, MS/MS (tandem MS), or Q-TOF. MALDI-TOF-MS can identify a wide mass range (1–300 kDa) of proteins in lymphocytes with a high accuracy and sensitivity and analyse post-translational protein modifications [64]. In combination with others, MALDI-TOF-MS is particularly suitable for the identification of protein spots via mass fingerprint or microsequencing. Peptide mass fingerprinting (PMF) by MALDI-MS and sequencing by tandem MS have evolved into the major methods for identification of protein following separation by two-dimensional gel electrophoresis, sodium dodecyl sulfate polyacrylamide gel electrophoresis, or liquid chromatography. The PMF for protein identification is exemplified with high molecular masses, low molecular masses, splice variants, aggregates with disulfide bridges, and phosphorylated proteins [65]. It is necessary to quantitatively determine the changes of proteomes or sub-proteomes in responses to stimuli and to fully elucidate lymphocyte signaling mechanisms and function. Isotope-coded affinity tagging is one of the most employed chemical isotope labeling methods and the first quantitative proteomic method to be based solely on using MS [66,67], and has been applied to a wide range of biological systems, including lymphocyte signaling [34,68]. By labeling cysteine residues of proteins from two different sources with ^12^C and ^13^C, respectively, isotope-coded affinity tagging allows comparison and relative quantitation of two or more bio-samples.

### Therapeutic significance

Proteomic approaches can determine expressed proteins in lymphocytes and describe the interactions between proteins and targeted regulatory signaling networks. Lymphocytes are a central component of immune defense mechanisms against pathogens and novel therapeutic strategies towards diseases. Identified profiles of tyrosine phosphorylation [31], proliferation [13], cell activation [37], or chromatin-associated proteins [34] in lymphocytes can help to build a comprehensive view of therapeutic strategies for diseases. CD20 and CD23 are immune regulators as therapeutic targets for heamatological malignancies, autoimmune diseases and allergic disorders, and a new subset of palmitoylated proteins in B cells [69].

## Conclusions

The understanding of lymphocyte profiles is one of the most critical approaches to investigate their functions and roles in the development of diseases. The proteomics-based studies on lymphocytes headlight the proteomic profiles of lymphocytes in diseases, such as autoimmune diseases, allergic inflammation and cancer, with a special focus on lung diseases. Proteomic studies of lymphocytes provide the potential significance of disease-specific diagnostic biomarkers and therapeutic targets. There is an urgent need of standard protocols to carry out proteomic studies on lymphocytes. The current achievements in profiling protein expression in lymphocytes will open the new window to develop therapeutic strategies and targets.

## Abbreviations

IL: Interleukin; VKH: Vogt-Koyanagi-Harada; IFN: Interferon; DRC: DNA repair capacity; HSP: Heat shock protein; IPF: Idiopathic pulmonary fibrosis; PPL: periplakin; MS: Mass spectrometry; MALDI: Matrix-assisted laser desorption/ionization; Q-TOF: Quadrupole time-of-flight; PMF: Peptide mass fingerprinting.

## Competing interest

The authors declare that they have no competing interests.

## Authors’ contributions

JBZ and ZTZ contributed to collection of information, analysis and interpretation of data and writing of the manuscript. CXB contributed to revision of the manuscript. HZS and XDW contributed to design and revision of the manuscript. All authors read and approved the final manuscript.
